# The Three-Y and Trachea View (3Y + T): A Conceptual Extension of the Three-Vessel and Trachea View for Comprehensive Fetal Echocardiographic Assessment

**DOI:** 10.3390/diagnostics16142180

**Published:** 2026-07-13

**Authors:** Ana M. Teixeira, Luís Guedes-Martins

**Affiliations:** 1 Instituto de Ciências Biomédicas Abel Salazar, University of Porto, 4050-313 Porto, Portugal; am.teixeiras@gmail.com; 2 Unidade Local de Saúde de Lisboa Ocidental, Hospital de Santa Cruz, 2794-035 Carnaxide, Portugal; 3 Centro de Medicina Fetal, Medicina Fetal Porto—Centro Materno Infantil do Norte, 4099-001 Porto, Portugal; 4 Unidade Local de Saúde de Santo António EPE, Centro Materno Infantil do Norte, Serviço de Obstetrícia, Departamento da Mulher e da Medicina Reprodutiva, Largo Prof. Abel Salazar, 4099-001 Porto, Portugal; 5 Unidade de Investigação e Formação—Centro Materno Infantil do Norte, 4099-001 Porto, Portugal; 6 Instituto de Investigação e Inovação em Saúde, Universidade do Porto, 4200-319 Porto, Portugal

**Keywords:** three-vessel view, three-vessel and trachea view, fetal echocardiography, prenatal diagnosis, congenital heart disease, aortic isthmus

## Abstract

**Introduction**: Congenital heart disease remains the most common congenital anomaly and a leading cause of infant morbidity and mortality. Although the three-vessel and trachea view has substantially improved prenatal assessment of the great arteries and aortic arch anatomy, detailed evaluation of the distal arch, aortic isthmus, and arch convergence may remain challenging in routine fetal echocardiography. **Methods**: A structured literature review of PubMed-indexed studies was conducted to examine the anatomical basis, clinical applications, diagnostic performance, and technological developments related to the three-vessel and trachea views. The reviewed evidence served as the scientific basis for the development of the proposed Three-Y and Trachea concept. **Results and Discussion**: The three-vessel and trachea view provides a standardized transverse assessment of the great vessels and contributes substantially to the prenatal detection of conotruncal and aortic arch anomalies. Building on this established approach, the proposed Three-Y and Trachea concept emphasizes the Y-shaped convergence of the ductal arch and transverse aortic arch at the level of the aortic isthmus, while preserving the trachea as a key anatomical landmark. This additional anatomical perspective may facilitate assessment of arch continuity, aortic isthmus morphology, and flow relationships within the upper mediastinum while highlighting the anatomical and hemodynamic significance of the aortic isthmus within the fetal circulation. **Conclusions**: The Three-Y and Trachea view represents a conceptual extension of the conventional three-vessel and trachea view that may offer a more comprehensive framework for anatomical and functional evaluation of the fetal aortic arch and aortic isthmus. Further prospective studies are required to assess its feasibility, reproducibility, and potential clinical value in prenatal cardiac screening.

## 1. Introduction

Congenital heart disease (CHD) is the most common structural congenital anomaly and remains a leading cause of infant morbidity and mortality in childhood. Its prevalence is approximately 8–9 per 1000 live births worldwide [1,2]. Accurate prenatal diagnosis is essential for optimizing perinatal and neonatal management and improving outcomes in affected fetuses and newborns [3,4].

Over the past decades, fetal echocardiography (FE) has become the cornerstone of prenatal cardiac assessment, enabling increasingly accurate detection of CHD and facilitating earlier diagnosis of severe cardiac malformations and fetal arrhythmias [5,6,7]. Historically, routine obstetric cardiac screening focused primarily on the four-chamber view (4CH), which reliably detects many intracardiac abnormalities, including severe ventricular hypoplasia and atrioventricular anomalies, but may fail to identify a substantial proportion of conotruncal and outflow-tract defects [1,8]. Allan et al. reported that approximately 60% of severe cardiac anomalies could be identified using the 4CH view alone [1,9]. Consequently, fetal cardiac screening has evolved from a predominantly 4CH-centered approach toward structured protocols incorporating outflow-tract assessment and transverse mediastinal planes [1,10,11,12]. Current professional recommendations support the routine inclusion of the 4CH view, ventricular outflow tracts, and transverse vessel views to improve the prenatal detection of clinically significant CHD [1,3,7].

A major advance in this evolution was the introduction of the three-vessel view (3V view) by Yoo et al., which provided a transverse cross-sectional assessment of the main mediastinal vessels and improved recognition of abnormalities involving the ventricular outflow tracts and great arteries [13,14,15]. However, this approach did not incorporate evaluation of the aortic arch (AoA) and its relationship to the trachea (T), an aspect that remains one of the most technically challenging components of fetal cardiac examination [8]. To address this limitation, the three-vessel and trachea (3VT) view was developed, allowing simultaneous visualization of the pulmonary artery (PA), ductus arteriosus (*DA*), upper aortic arch (AoA), superior vena cava (SVC), and trachea (T) within a single transverse plane [12,14]. The incorporation of the 3VT view into routine fetal cardiac assessment provided a rapid, accessible, and reproducible method for evaluating the upper mediastinum and significantly improved detection of abnormalities involving the outflow tracts, AoA, and great vessel arrangement [1,12,14,16,17]. As a result, the 3VT view has become an integral component of contemporary prenatal cardiac screening and is now included in several standardized examination protocols [1,3]. Additional refinements, including the simplified five-transverse-plane approach proposed by Li et al., have further strengthened the role of transverse mediastinal imaging in systematic fetal cardiac evaluation [18].

The 3VT plane has evolved beyond a simple screening tool and now represents one of the most informative transverse views in FE. By enabling rapid assessment of vessel number, size, alignment, and spatial relationships, while simultaneously facilitating Doppler evaluation of blood flow patterns, the 3VT view contributes substantially to the prenatal detection of major CHD [14,15]. Updated practice guidelines continue to support the incorporation of 3V and 3VT imaging into routine fetal cardiac screening because of their demonstrated value in improving diagnostic performance beyond reliance on the 4CH view alone [1,7].

The aim of this article is to review the anatomical and clinical foundations of the 3VT view and to introduce the proposed Three-Y and Trachea (3Y + T) concept as a potential extension of upper mediastinal fetal cardiac imaging. The proposed concept is intended to emphasize the anatomical relationship between the *DA*, transverse aortic arch (AoT), aortic isthmus (AoI), and descending aorta (DAo) while maintaining the trachea as a central reference landmark. By integrating structural and Doppler-based functional assessment into a single imaging plane, the 3Y + T concept may facilitate evaluation of selected aortic arch and ductal abnormalities. Its clinical applicability and diagnostic performance remain to be established through future prospective validation studies.

## 2. Methods

A structured literature search was performed using the PubMed database to support this narrative review and to identify studies addressing the anatomical basis, diagnostic performance, and hemodynamic assessment of the 3V and 3VT views in FE. The initial search was performed in October 2025, and the final update was completed in July 2026 to ensure inclusion of the most contemporary evidence available. The search strategy was optimized through the use of controlled vocabulary from the Medical Subject Headings (MeSH) Database, incorporating the descriptors “Fetal Heart,” “Fetus,” and “Ultrasonography, Prenatal,” alongside more specific terms widely employed in the literature, including “three-vessel”, “three-vessel view” and “three-vessel and trachea.” Additional keywords targeted the cardiovascular structures fundamental to the assessment of the 3V and 3VT views, such as “Pulmonary Artery,” “Aorta,” “Superior Vena Cava,” “Great Vessels,” “Mediastinum,” “Trachea,” and “Reference Values,” combined with hemodynamic terms such as “Echocardiography AND Doppler,” “Doppler Blood Flow,” and “Blood Flow Velocity”. Additional strategies were used to improve the comprehensiveness of the search, including the PubMed Advanced Search Builder, which enabled the combination of free-text terms such as “three-vessel view,” “three-vessel and trachea view,” “fetal echocardiography,” “prenatal diagnosis,” and “congenital heart disease”.

**Figure 1:**
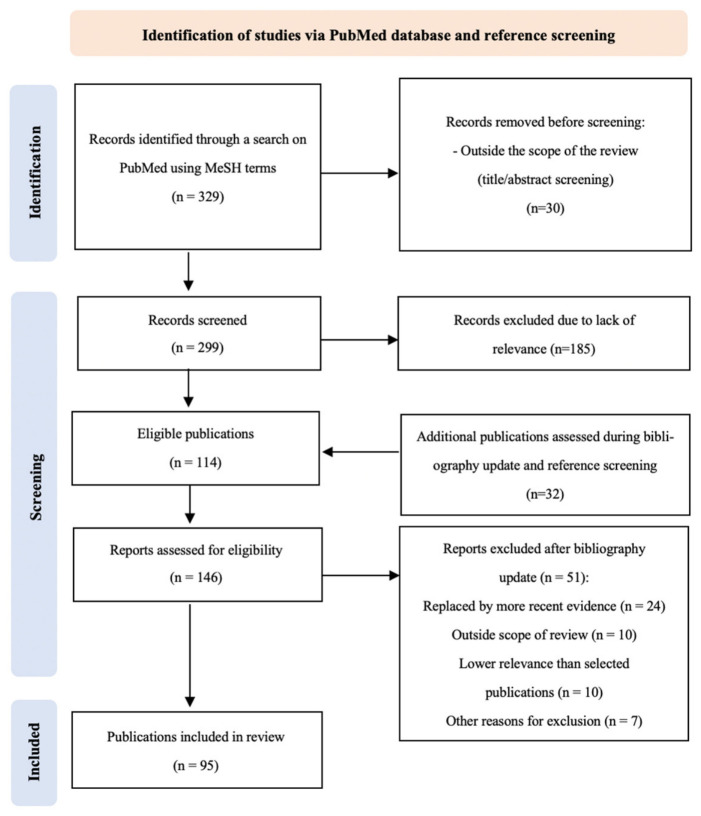
Study selection flow diagram.

The reference lists of relevant articles were examined, and PubMed’s “Similar Articles” function was utilized to identify additional studies not captured by the structured search. The literature search and study selection process were informed by the PRISMA 2020 framework to enhance transparency in reporting. However, the present work was designed and conducted as a narrative review and should not be interpreted as a systematic review or meta-analysis (Figure 1). Eligibility criteria included full access to methodological details and outcome data. Case reports and conference abstracts were excluded due to their limited scope and insufficient methodological rigor. Study selection followed a two-phase process. First, titles and abstracts were screened to identify potentially relevant studies. Subsequently, the full texts of all shortlisted articles were reviewed in detail to determine final inclusion or exclusion based on predefined criteria. As appropriate for a narrative review, formal independent duplicate screening and risk-of-bias assessment were not performed. Any uncertainties regarding study relevance were resolved through careful reassessment to ensure consistency with the predefined scope and objectives of the review.

## 3. Anatomical Basis of the 3VT View

Imaging of the great vessels is now widely accepted as a fundamental component of the standard obstetric ultrasound (US) examination and is included in all screening guidelines [1]. A thorough understanding of great vessel anatomy and the relevant US views is essential for performing an effective obstetric US examination [1,14]. Incorporating outflow tract and upper mediastinal views into routine fetal cardiac assessment has significantly improved prenatal detection of CHD, particularly conotruncal anomalies and AoA abnormalities [1,7]. In addition to the standard 4CH view, the 3V and 3VT views provide essential insights into the alignment, size, and spatial relationships of the great arteries and the SVC within the upper mediastinum [12,14,16]. The 3VT view, in particular, uses the trachea as a stable central reference to evaluate aortic sidedness and the configuration of the *DA* [1]. A comprehensive understanding of normal anatomy and its variants within these planes is essential for accurate screening and to avoid overdiagnosis [19].

### 3.1. Normal Anatomy of the Great Vessels in the Upper Mediastinum

**Figure 2:**
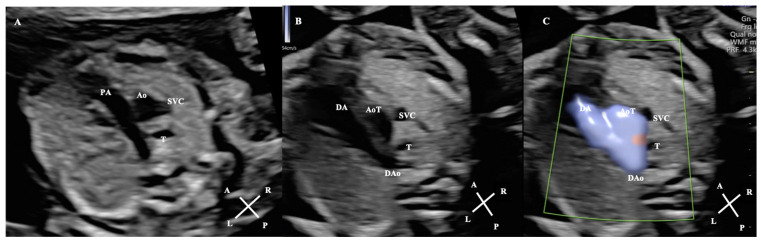
The standard 3V view depicts a transverse echocardiographic plane of the fetal thorax showing three vascular structures in cross-section: from left (L) to right (R), the main pulmonary artery (PA), the ascending aorta (Ao), and the superior vena cava (SVC). These vessels form a gentle curve: the PA is anterior (**A**) and L, the Ao is more posterior (P) and centrally positioned, and the SVC is P and R. In a healthy heart, the PA or *DA* is typically slightly larger than the Ao, and both are larger than the SVC (**A**). The 3VT view (**B**) demonstrates the AoT and the *DA* converging toward the descending aorta (DAo), forming a characteristic “V-shaped” configuration to the left of the trachea (T). The *DA*, which is slightly larger and more A, arises from the PA and joins the DAo. The trachea appears as an echogenic ring with a hypoechoic lumen located posterior to the arteries. In normal anatomy, both arches are positioned to the L of the T, creating a left-sided “V” in the 3VT view. The corresponding image with color Doppler illustrates a normal heart, showing antegrade, laminar flow in both the AoT and *DA*, directed posteriorly toward the DAo (**C**).

In the standard 3V view, three vascular structures appear in cross-section: from left to right, the main PA (or its continuation as the *DA*), the ascending/transverse aorta, and the SVC [13,19]. The three vessels form a gentle curve, with the PA most anterior, followed on the left by the aorta (Ao), which is more posterior and central, and the SVC posterior and to the right [1,3] (Figure 2A). A key feature is their relative size. In a healthy heart, the PA (or *DA*) is typically slightly larger than the Ao, and both are larger than the SVC [8,14]. This consistent anatomical arrangement and proportional size relationship indicate normal ventriculo-arterial connections and unobstructed flow through both the right and left ventricular outflow tracts. In the 3VT view, the AoT and the *DA* converge toward the DAo, forming a characteristic “V-shaped” configuration to the left of the trachea [8,12,19]. The *DA* is typically more anterior and slightly larger, arising from the PA and joining the DAo, whereas the AoA originates from the ascending aorta (AAo), courses posteriorly and to the left, and likewise continues as the DAo [1,8,14]. This arrangement, in which both arches course to the left of the trachea and merge into a single DAo, is the hallmark of normal fetal upper mediastinal anatomy [1,7]. The 3V and AoA views are used to assess the size and spatial arrangement of the great arteries, providing additional diagnostic clues for abnormalities involving the ventriculo-arterial connections [1,8,13,14].

### 3.2. Relationship Between Pulmonary Artery (PA), Aorta (Ao), Superior Vena Cava (SVC), and Trachea (T)

The trachea is a central, relatively fixed structure in the upper mediastinum. In the US, it appears as an echogenic ring encircling a hypoechoic lumen, located behind the great arteries [1,3,12]. Its position serves as a reliable reference point for assessing the sidedness and relative positions of the ductal and aortic arches. Normally, both arches are expected to run to the left of the T, creating a left-sided “V” pattern in the 3VT view [1,3,14]. In a typical *situs solitus* with a left-sided aortic arch, the *ductus arteriosus* (*DA*) runs from the main pulmonary artery (PA) to the left and behind, connecting with the ductal arch (DAo) [1,3,14]. The aortic arch (AoA) arises from the ascending aorta (AAo), passes in front of the trachea and esophagus, then curves leftward and backward [1,14]. In the 3VT section, the transverse segments of these arches are observed to the left of the T, converging into the DAo [1,16]. This left-sided relationship serves as a key reference point: any right-sided configuration of an arch or a “U-shaped” formation around the trachea indicates variants such as a right aortic arch (RAA) or vascular rings [1,20]. In the 3VT view, the pulmonary artery (PA) and ductus arteriosus (*DA*) are usually the most anterior structures [3,14,21]. The aortic arch (AoT) lies slightly posterior and to the right of the ductus, while remaining to the left of the trachea [1,3]. The superior vena cava (SVC) is visualized as a smaller vessel positioned to the right and posterior to the aorta, draining into the right atrium [1,20]. This consistent anterior-to-posterior layering (PA/*DA* anterior, Ao intermediate, SVC posterior) and right-to-left alignment (PA, Ao, and SVC from left to right) are essential criteria for confirming normal mediastinal vascular anatomy and for distinguishing it from abnormalities such as transposition of the great arteries (TGA), double-outlet right ventricle (DORV), or arch anomalies [1,14,17]. At the 3VT level, the thymus is best visualized in this transverse view of the upper mediastinum, appearing as a hypoechoic structure between the sternum and the great vessels, and clearly delineated from the lung [22,23]. By assessing the 3VT relationships among the PA, Ao, SVC, and T, sonographers can rapidly identify changes in vessel size, alignment, and sidedness that are suggestive of congenital heart disease (CHD) [1,3,14,24]. Several studies have demonstrated that incorporating the 3V/3VT views into routine screening significantly improves the detection of conotruncal and arch anomalies compared with reliance on the 4-chamber (4CH) view alone [1,15,17]. However, this advantage depends on a thorough understanding of normal anatomy and its variants to avoid misinterpreting benign variants as pathological conditions [25].

### 3.3. Variants of Normal: Laterality and Axis Considerations

In a typical fetus, *situs solitus* features the heart apex pointing left, the stomach and spleen on the left, and the liver primarily on the right [1,3]. The fetal cardiac axis in a 4CH view usually lies about 45° to the left of the midline, with a normal range of 25–65° [1,3]. A slight shift in the axis and the position of the heart within the chest can subtly alter the appearance of the 3V and 3VT views [1]. However, as long as the relative positions of the PA, Ao, SVC, and T remain consistent, these differences are considered normal variants rather than indicators of structural disease [1,3,7,12]. Quantitative evaluation of the ductal and aortic arches in the 3VT plane has enabled the development of reference ranges and z-scores specific to different gestational ages [26]. In uncomplicated pregnancies, both the AoI and the *DA* gradually grow with gestation. Typically, the ductus remains slightly larger than the AoA and joins the left side of the trachea in a well-organized V-shaped formation [1,12]. Understanding these normal ranges is important, as small differences in vessel size or slight angular variations may represent normal anatomical variability and do not necessarily indicate developing coarctation or outflow tract obstruction [3,26]. Although the normal fetus typically has a left-sided AoA, a RAA may occur as an isolated variant or in association with vascular rings and a genetic syndrome [1,14,27]. In the 3VT view, a RAA that does not form a complete vascular ring is visualized as an arch coursing to the right of the T. The *DA* connects to the DAo on either the same or the opposite side, depending on the branching pattern [28,29]. Careful evaluation of the arch pathways in relation to the T, together with assessment of systemic and pulmonary venous return and extracardiac structures, is essential for distinguishing a “benign” isolated RAA from ring-shaped configurations such as a double aortic arch (DAA) or aberrant subclavian arteries [28,30]. Aberrant right subclavian artery (ARSA) is a common anomalous branch of the AoA and can often be detected prenatally with detailed examination [31,32,33]. In upper mediastinal views, ARSA arises distal to the AoA and courses posterior to the trachea and esophagus toward the right upper limb, typically best visualized in a slightly oblique plane [14]. Although ARSA is frequently isolated and hemodynamically insignificant, it may act as a soft marker for trisomy 21 and other chromosomal abnormalities [31]. Therefore, it is regarded as a “variant” that may appear structurally benign but carries important genetic and counseling implications [31,33].

Disorders of laterality, such as situs inversus and heterotaxy, markedly alter the typical configuration of the great vessels in the upper mediastinum [3]. In these cases, the 3V and 3VT views should be interpreted in the context of the overall situs, because factors such as the heart’s orientation, atrial position, abdominal organ placement, and venous anomalies can alter the usual spatial relationships used as references [3]. In particular, interruption of the inferior vena cava with azygos continuation is a characteristic finding in left atrial isomerism and may be an important diagnostic clue to heterotaxy. Left atrial isomerism is also frequently associated with congenital atrioventricular block and fetal bradycardia, underscoring the importance of integrating structural and rhythm assessment during fetal echocardiography [34]. Although a detailed segmental analysis remains important, understanding normal upper mediastinal anatomy helps the examiner identify deviations from typical variants that suggest a more complex laterality disturbance [3].

The 3V and 3VT views are now recognized as essential components of a comprehensive fetal cardiac assessment and are endorsed by leading organizations such as the International Society of Ultrasound in Obstetrics and Gynecology (ISUOG) and the American Institute of Ultrasound in Medicine [1,7]. These planes enable simultaneous evaluation of great artery alignment, relative vessel size, and AoA sidedness relative to the T, thereby improving prenatal detection of conotruncal, AoA, and vascular ring anomalies [14]. A thorough understanding of normal anatomy, physiological variation, and benign variants, particularly those related to the cardiac axis, arch dimensions, and branching patterns, is crucial for minimizing false-positive diagnoses and avoiding unnecessary parental anxiety [1,3,14]. Integrating systematic assessment of the 3V/3VT views into routine obstetric US examinations, together with timely referral for formal FE when abnormalities are suspected, is essential for optimizing prenatal cardiac screening [1,3].

## 4. Technique and Acquisition

### 4.1. Transducer Orientation and Sweep from the Outflow Tract

The sonographic technique involves performing a transverse sweep, moving the transducer upward from the 4CH view toward the fetal upper mediastinum [1,3]. This method allows a step-by-step assessment of cardiac structures and provides the necessary views to confirm the normality of the outflow tracts and vessels [3,19]. Usually, the outflow-tract and great-vessel views are obtained by directing the transducer toward the fetal head (sweep technique) with slight angle adjustments, starting from a 4CH view, to visualize the origins of the Ao and PA as they cross [1,3]. Additionally, the PA bifurcation is visible [3]. The detailed acquisition steps start with the 4CH view. This step helps visualize both arches clearly without distortion, ensuring an accurate representation of their structural relationship [3]. Different angles and positions of the transducer are crucial for distinguishing normal variations from critical pathological abnormalities in the great vessels [1,3]. As the transducer progresses, the spatial relationships among the Ao, *DA*, and T form a key diagnostic configuration [1]. All structures, such as the Ao, *DA*, SVC, and T, must be clearly visible and properly oriented [1,3]. This detailed examination helps identify any irregularities or deviations from the normal, which are vital for prenatal diagnosis and planning. By adopting this systematic approach, clinicians can improve the accuracy and effectiveness of fetal echocardiographic assessments, thereby enabling early detection and appropriate management of vascular anomalies [14,19].

### 4.2. Optimization Strategies for 2D and Color Doppler Imaging

FE involves capturing anatomical planes such as the 4CH, left ventricle outflow tract (LVOT), and right ventricle outflow tract (RVOT), and carefully adjusting imaging settings to ensure high diagnostic accuracy [1,3]. ISUOG guidelines recommend systematically including additional transverse planes, such as the 3VT view, as this enhances the detection of cardiac and vascular anomalies when used with proper imaging and Doppler settings [1]. Accurately identifying the 3VT plane first depends on correctly establishing fetal situs. This step is crucial for guiding subsequent cardiac evaluations and reducing errors in anatomical interpretation [3]. Optimizing the two-dimensional (2D) image in the 3VT plane requires specific adjustments to the transducer and machine settings [1,3]. Transducer frequency selection should balance resolution and penetration: higher frequencies are beneficial in early gestation to enhance superficial detail, whereas lower frequencies are needed for deeper fetal positions [1,3,14,35]. Reducing the sector width and depth to focus on the upper mediastinum greatly improves temporal and lateral resolution, resulting in higher frame rates and sharper visualization of blood vessels [3]. Using harmonic imaging and adjusting dynamic compression enhances the contrast between vessels and surrounding tissues, helping to better define vascular outlines and evaluate spatial relationships [3]. Detecting subtle anatomical differences associated with CHD is essential [1,3,16]. Following 2D echocardiography, color Doppler is essential for detecting functional abnormalities. It significantly improves exam sensitivity by allowing visualization of regurgitation jets, valvular stenosis, and reversed flow in major vessels [3,36]. Adding the color Doppler to the 3VT view provides essential hemodynamic information, identifying flow changes that are not visible on 2D imaging [1,3]. Optimal color Doppler settings are particularly important when evaluating the spatial relationship and flow continuity between the ductal arch and AoT within the 3VT plane. Precise visualization of the convergence between the *DA*, AoT, and DAo is essential for accurate assessment of upper mediastinal anatomy and forms the anatomical basis for the proposed 3Y + T concept.

### 4.3. Pitfalls and Common Artifacts

Interpretation of the 3VT view in FE can be influenced by technical factors, such as transducer angulation and color Doppler settings, as well as by temporary anatomical variations, such as fetal position and gestational age. These factors often lead to false positives or negatives when diagnosing CHD. Physiological changes, such as transient differences in the ventricles or major vessels, can mimic coarctation of the aorta (CoA), especially in the second trimester, leading to overdiagnosis [27]. Additionally, difficulty distinguishing the AoA from the *DA* on oblique views can create a false impression of arch interruption or abnormal vessel alignment [14]. Technical artifacts affect the accuracy of interpreting the 3VT plane. Improper color Doppler use, such as excessive gain, an incorrect velocity scale, or poor wall filter adjustment, can cause aliasing, color bleeding, and blurry boundaries, mimicking turbulent flow or false stenosis [1,3,37,38].

**Figure 3:**
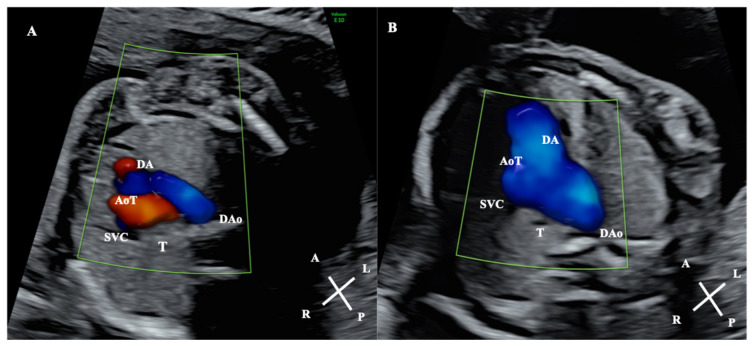
The standard 3VT view with color Doppler demonstrates the fetal thorax in a transverse plane. An incorrect Doppler angle, positioned perpendicular to the direction of flow, may create a misleading appearance of critical aortic obstruction by suggesting retrograde flow in the transverse aortic arch (AoT). From left to right: ductal arch (*DA*), AoT with apparent retrograde flow, and superior vena cava (SVC) (**A**). When the same image is evaluated using an appropriate Doppler angle, a normal 3VT view is observed, with antegrade, laminar flow in both the AoT and *DA*, directed posteriorly toward the descending aorta (DAo) (**B**).

Perpendicular insonation decreases Doppler sensitivity, which can conceal vascular segments and cause false indicators of interruption [1,3,14,35] (Figure 3). Limited spatial resolution in fetuses with difficult positions, maternal obesity, or oligohydramnios makes it harder to differentiate normal variants from pathology. Guidelines advise correlating the 3VT with other cardiac views and re-assessing when in doubt to minimize errors and enhance accuracy [1,3].

## 5. The 3VT View as a Screening Tool

### 5.1. How the 3VT View Improves the Detection of Conotruncal Anomalies

FE is a crucial screening technique for assessing the fetal heart, with the 3VT view now recognized as a vital part of the standardized transverse scanning protocol [1,3,7]. The traditional emphasis on the 4CH view has evolved, as the incorporation of outflow tract and upper mediastinal views, including the 3VT view, substantially improves prenatal detection of major CHD compared with the 4CH view alone [1,3,16]. Current professional guidelines mandate the inclusion of the 3VT view in both grayscale imaging and digital documentation, as it facilitates straightforward identification of abnormalities in the ventricular outflow tracts and great arteries during routine screening [3,7]. This view is particularly effective for detecting conotruncal anomalies, a frequent cause of cyanotic CHD, which may be overlooked in routine assessments because they can appear normal on the 4CH view [39,40]. In clinical practice, the 3VT view is used to evaluate the caliber and anatomical relationships of the great vessels and arches, whereas color Doppler provides additional functional information on flow direction and vessel patency [1,14,36]. Furthermore, implementing a comprehensive screening protocol that regularly assesses anatomy and hemodynamics at the 3VT level, often using color Doppler, substantially improves the detection of structural defects compared with traditional methods [10,37]. Specifically, the 3VT effectively detects conotruncal defects such as Tetralogy of Fallot (TOF), TGA, DORV, and AoA anomalies, which are identified by unusual vessel connections or the lack of the typical *DA* confluence [38,39,40]. For example, a “U-shaped” confluence at the 3VT level indicates a RAA, with the trachea and esophagus located between the two arches instead of on the right side [28,38]. Using color Doppler at this level can also help identify significant hemodynamic issues [1,3,14,35].

Overall, the 3VT view is a vital part of fetal cardiac screening, improving detection of complex heart defects that could otherwise be overlooked [27]. It is especially effective for detailed lesions such as TGA, TOF, and AoA anomalies [3,12]. In clinical practice, these perspectives are highly interconnected and should be evaluated together. For instance, diagnosing conotruncal defects based solely on the 3V is unlikely without examining the AoT, which is best visualized on 3VT with slight transducer adjustments [12,14]. Furthermore, when evaluating 3VT in cases of CHD, operators can often visualize the PA bifurcation, which enhances confidence in identifying ductal or AoA anomalies [12].

### 5.2. Diagnostic Clues: Abnormal Number, Size, or Arrangement of Vessels

The 3VT view allows systematic evaluation of several anatomical and hemodynamic parameters that provide diagnostic information. Usually, the fetal PA, Ao, and SVC are aligned in a straight line from the left anterior to the right posterior [1].

**Figure 4:**
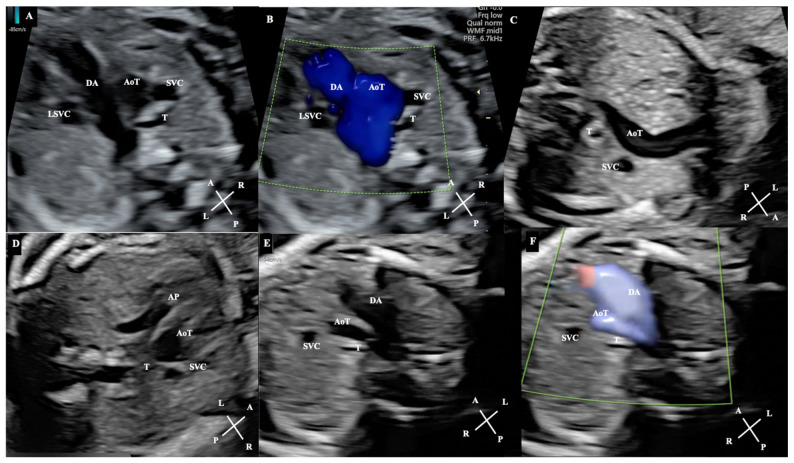
Echocardiographic transverse plane of the fetal thorax at the level of the classic 3VT view. From left (L) to right (R), the ductal arch (*DA*), the transverse aortic arch (AoT) passing to the right of the trachea (T), and the right superior vena cava (SVC) are identified. This view demonstrates a supernumerary vessel on the left side of the Pulmonary Artery (PA) and the *DA*, suggestive of a persistent left superior vena cava (PLSVC). From L to R, the vessels visualized are the LSVC, the *DA*, the AoT, and the right SVC. This configuration represents a “four-vessel” rather than a three-vessel view, shown in a 2D image (**A**) and with color Doppler (**B**). In a fetus with Transposition of the Great Arteries (TGA), only two vessels are visualized instead of three. From L to R, these are the AoT and the SVC. The AoT demonstrates rightward convexity as it arises from the anterior right ventricle (RV) (**C**). In a fetus with Tetralogy of Fallot (TOF), this view shows a smaller pulmonary artery (PA) and an enlarged AoT (**D**). In a fetus with Aortic Coarctation (CoA), a discrepancy in vessel size is observed, with a larger PA and a smaller AoT shown in a 2D image (**E**) and with color Doppler (**F**).

These vessels should come together to form a V-shaped junction positioned to the left of the outflow tract [1]. A U-shaped confluence encircling the outflow tract and esophagus is a common indication of a RAA [28,30]. Conversely, a RAA associated with a right *DA* produces a right-sided V-sign, rather than the characteristic U-sign, representing an uncommon anatomical variant that should be recognized during assessment of the 3VT view because of its specific associations and implications for prenatal counseling [41]. Additionally, when the great arteries lose their normal crossing pattern and appear parallel to one another, this is a significant sign of TGA or DORV [42,43,44]. An abnormal number of mediastinal vessels is also indicative of various conditions [14]. A supernumerary vessel on the left side of the PA often suggests a persistent left superior vena cava (PLSVC) [1,14].

(Figure 4A,B). In contrast, the presence of a single large vessel leaving the heart, as opposed to separate aortic and pulmonary arteries, indicates truncus arteriosus or a common arterial trunk (CAT) [1,12,14]. Blood vessels typically follow a size hierarchy, with the PA being the largest, followed by the AAo and then the SVC. A notably larger Ao compared to the thinner PA is a common feature in TOF. On the other hand, if the AoA is smaller than the *DA*, it could suggest AoA hypoplasia or coarctation [14]. This plane also offers hemodynamic insights. Color Doppler in the 3VT view is essential for verifying antegrade flow and consistency across both arches [3]. Retrograde flow in either arch suggests significant pathology; specifically, reverse flow in the AoA may indicate aortic valve atresia or coarctation, whereas reverse flow in the *DA* may indicate pulmonary valve stenosis or atresia [10,37,38]. Although this method is highly effective, it is important to note that a normal 3VT view does not exclude all conotruncal anomalies, as some cases of DORV or corrected transposition of the great arteries may appear normal [42,43,44].

### 5.3. Integration into Routine Mid-Trimester Scans

The 3VT view has become an integral component of contemporary mid-trimester fetal cardiac screening. Because it can be obtained through a simple cranial sweep from the 4CH view, it represents a practical and reproducible addition to routine obstetric US examinations without substantially increasing examination time [15]. Its incorporation into standardized screening protocols allows for systematic assessment of great-vessel arrangement, AoA sidedness, and upper mediastinal anatomy within a single transverse plane [14]. International guidelines, including those issued by ISUOG and other professional organizations, recommend the routine evaluation of transverse mediastinal planes as part of a comprehensive fetal cardiac examination [1]. The inclusion of the 3VT view complements the 4CH and outflow-tract views by providing additional information on the spatial relationships among the great vessels and the T, thereby increasing diagnostic confidence during prenatal cardiac screening [1,14]. The widespread adoption of the 3VT view reflects its feasibility, reproducibility, and clinical utility in routine practice. Accurate interpretation, however, depends on adequate operator training and familiarity with normal anatomical variants, thereby reinforcing the importance of continued education and quality assurance in fetal cardiac screening programs [45].

## 6. Key Congenital Heart Defects Diagnosed via the 3VT View

### 6.1. Transposition of the Great Arteries (TGA)

The 3VT view provides important diagnostic clues for the prenatal detection of TGA, one of the most common cyanotic CHD. In contrast to the normal appearance of three mediastinal vessels, fetuses with TGA often demonstrate only two vessels within the 3VT plane—the Ao and the SVC—giving rise to the characteristic “misnomer three-vessel” sign [43]. This finding reflects an abnormal spatial relationship between the great arteries and should prompt a detailed evaluation of the ventricular outflow tracts. Another useful sonographic marker is the abnormal rightward convexity of the AAo as it arises from the anterior right ventricle, in contrast to the normal leftward curvature observed in healthy fetuses [46] (Figure 4C). When combined with outflow-tract imaging and the five-chamber view, the 3VT view improves recognition of the parallel course of the great arteries and facilitates differentiation from normal ventriculo-arterial anatomy [43,46]. Although a definitive diagnosis requires a comprehensive FE assessment, the 3VT view remains a valuable screening tool for identifying abnormal vessel arrangement and raising early suspicion of TGA, thereby facilitating prenatal counseling, delivery planning, and postnatal management [43].

### 6.2. Tetralogy of Fallot (TOF) and Pulmonary Atresia (PAtr.)

The 3VT view is particularly valuable for the prenatal identification of TOF and pulmonary atresia with ventricular septal defect (PA-VSD), as both conditions produce characteristic alterations in the size and spatial relationships of the great vessels [1,3,47]. In fetuses with TOF, the 3VT view typically demonstrates a relatively small PA and an enlarged AoA, reflecting aortic override and reduced pulmonary outflow [47] (Figure 4D). This abnormal vessel-size relationship is often readily recognizable in the upper mediastinum and represents an important screening clue. In PA-VSD, the PA may be severely hypoplastic or absent, resulting in marked distortion of the normal three-vessel configuration [40,48]. The 3VT view facilitates recognition of these abnormalities by providing simultaneous visualization of the great vessels and their relative calibers. Color Doppler assessment may further assist in characterizing pulmonary blood flow and identifying associated abnormalities of the ductal circulation [3,35]. By highlighting abnormalities in vessel size, alignment, and flow patterns, the 3VT view contributes substantially to the prenatal detection of TOF spectrum disorders and supports early referral for comprehensive fetal echocardiographic assessment [1,3,14].

### 6.3. Coarctation of the Aorta (CoA) and Interrupted Aortic Arch (IAA)

**Figure 5:**
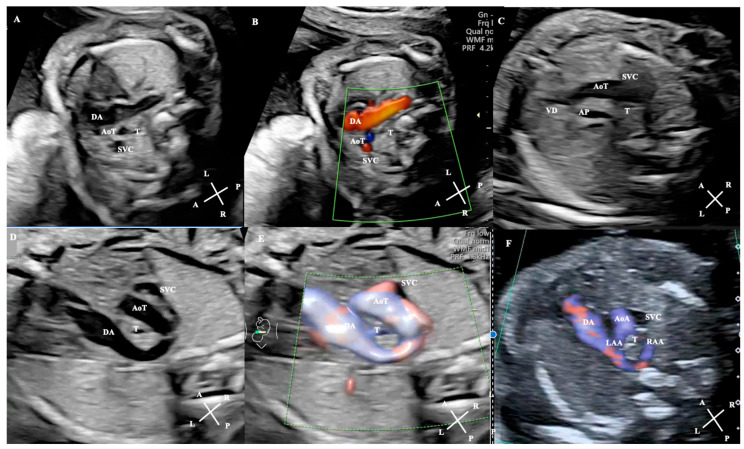
Echocardiographic transverse plane of the fetal thorax at the level of the classic 3VT view. In a fetus with Interruption of the aortic arch (IAA), the view demonstrates absence or discontinuity of the AoT, or a small aorta (Ao). From left (L) to right (R), the vessels are the *DA*, the smaller Ao, and the SVC (**A**), and the same image with color Doppler (**B**). In a fetus with double-outlet right ventricle (DORV), with a subaortic ventricular septal defect, PA stenosis, and right aortic arch (RAA), the view shows, from L to R, the SVC, the smaller PA, and the larger RAA positioned to the right of the trachea (T) (**C**). In a fetus with a right-sided aortic arch (RAA) instead of the typical V-shape, a U-shaped confluence encircling the trachea and esophagus is observed (**D**). The corresponding color Doppler image is shown in (**E**). In a fetus with a double aortic arch (*DA*A), the view demonstrates, from L to R, the *DA*, the bifurcation of the aortic arch (AoA) into the left aortic arch (LAA) and the dominant right aortic arch (RAA) encircling the T, and the SVC (**F**).

The 3VT view is one of the most informative transverse planes for the prenatal assessment of CoA and interrupted aortic arch (IAA), as both conditions affect the morphology, continuity, and caliber of the AoA [49,50,51,52]. Within this plane, the relative sizes and spatial relationships of the PA, AoT, and *DA* can be evaluated simultaneously, providing important anatomical and hemodynamic clues. In fetuses with suspected CoA, the 3VT view typically demonstrates a discrepancy in vessel size, characterized by a relatively enlarged PA and a smaller AoT [49,50] (Figure 4E,F). Careful assessment of the AoA and AoI may reveal hypoplasia or narrowing, while color and spectral Doppler can identify abnormal flow patterns that further increase diagnostic suspicion [49]. Because CoA primarily affects the transition between the AoA and DAo, evaluation of the AoI is particularly important when interpreting abnormalities within the 3VT plane. In IAA, the normal continuity of the aortic arch is disrupted, resulting in the absence or interruption of the expected arch configuration within the upper mediastinum [53,54,55] (Figure 5A,B). The 3VT view facilitates recognition of these abnormalities by demonstrating discontinuity of the AoT, abnormal vessel proportions, or altered Doppler flow patterns. In both CoA and IAA, integration of grayscale and Doppler findings improves diagnostic confidence and helps identify fetuses who require detailed fetal echocardiographic evaluation and specialized perinatal management [51,52,54,55]. Because these lesions directly affect the continuity and caliber of the AoT, AoI, and DAo, they highlight the potential value of imaging approaches that facilitate more detailed evaluation of this anatomical region within the upper mediastinum.

### 6.4. Double-Outlet Right Ventricle (DORV)

The 3VT view provides important diagnostic clues in fetuses with DORV, a congenital anomaly characterized by abnormal ventriculo-arterial connections [56,57] (Figure 5C). Although definitive diagnosis requires detailed evaluation of the relationship between the great arteries and the ventricular septal defect (VSD), abnormalities of vessel size, orientation, and spatial arrangement are frequently apparent within the upper mediastinum [57]. In the 3VT plane, DORV may present with distortion of the normal great-vessel configuration, abnormal vessel relationships, or associated findings such as pulmonary stenosis and RAA [14,57]. Careful assessment of the great vessels and their relationship to the trachea can provide important diagnostic clues and facilitate differentiation from other conotruncal anomalies, including TOF and TGA [14,57]. Color Doppler imaging and advanced flow visualization techniques may further improve the characterization of vessel orientation and hemodynamic relationships, thereby supporting a more comprehensive prenatal assessment. Consequently, the 3VT view is an important component of the systematic echocardiographic evaluation of fetuses with suspected DORV and helps identify associated anomalies that may influence perinatal management and postnatal outcomes [3,57].

### 6.5. Right Aortic Arch (RAA) and Double Aortic Arch (DAA)

The 3VT view is particularly valuable for the prenatal detection of AoA laterality abnormalities and vascular rings because it directly demonstrates the relationships among the AoA, *DA*, and T [14,28,58,59]. In normal fetal anatomy, both arches course to the left of the T, forming the characteristic left-sided “V-sign”. Alteration of this relationship represents an important diagnostic clue. In fetuses with a right aortic arch (RAA), the AoT courses to the right of the trachea rather than to the left [14,28] (Figure 5D,E). Instead of the normal V-shaped configuration, the 3VT view frequently demonstrates a characteristic U-shaped arrangement surrounding the trachea and the esophagus, particularly when a vascular ring is present [28]. Recognition of this abnormal spatial relationship is one of the major strengths of the 3VT examination and facilitates differentiation from normal arch anatomy. In DAA, the AAo divides into right and left arches that encircle the trachea and esophagus, forming a complete vascular ring [58,59] (Figure 5F). The 3VT view allows simultaneous visualization of both arches and their relationship to the T, providing a distinctive imaging pattern that supports prenatal diagnosis and differentiation from other arch anomalies. Because both RAA and DAA are defined by abnormal arch laterality and vascular relationships, they represent conditions for which the 3VT view is particularly informative. Early recognition facilitates comprehensive fetal echocardiographic assessment, parental counseling, and appropriate perinatal planning [20,28,58,59].

### 6.6. Abnormal Laterality: Situs Ambiguous and Isomerism Syndrome

The 3VT view plays an important role in the prenatal assessment of laterality disorders, including situs ambiguous and isomerism syndromes, because it provides a rapid overview of the spatial relationships among the great vessels, AoA, and T [14,60,61]. Since these conditions are frequently associated with complex CHD and abnormal vascular anatomy, deviations from the expected mediastinal arrangement may represent an important diagnostic clue. In fetuses with heterotaxy syndromes, the 3VT view may demonstrate unusual vessel positions, abnormal arch sidedness, atypical vascular branching patterns, or disrupted relationships between the great vessels and the trachea [60,61]. Findings such as RAAs, DAAs, or abnormal venous connections may raise suspicion of an underlying laterality disorder and prompt more comprehensive segmental cardiac evaluation [14,60,61]. Although the diagnosis of situs abnormalities requires assessment of the overall fetal anatomy, including cardiac position, abdominal organ arrangement, and systemic and pulmonary venous return, the 3VT view provides a valuable screening perspective that facilitates recognition of abnormal mediastinal vascular patterns and supports the early identification of complex laterality disorders [3,14,60,61].

## 7. Advanced Applications

### 7.1. Use of Color and Power Doppler for Flow Direction and Velocity Assessment

The 3VT view provides a unique transverse perspective of the fetal upper mediastinum, allowing simultaneous assessment of the great vessels and their hemodynamic relationships [38,43,62,63,64]. The addition of color and power Doppler substantially enhances the diagnostic value of this plane by enabling real-time visualization of flow direction, vessel patency, and flow disturbances within the great arteries and *DA* [3,36,39,40,65]. Color Doppler helps identify abnormal blood flow patterns associated with CHD, such as turbulence, flow acceleration, and changes in flow direction due to valvular stenosis, atresia, or obstruction [3,54]. Power Doppler may further enhance the visualization of low-velocity blood flow and small vascular structures, complementing traditional color Doppler assessment. Additionally, Doppler interrogation of the *DA*, AoT, and AoI may provide valuable information on flow distribution and arch hemodynamics, especially in fetuses with suspected arch abnormalities [1,14,35,48]. Spectral Doppler offers quantitative assessment of blood-flow velocity and waveform morphology, contributing to the evaluation of stenotic, regurgitant, and ductal-dependent lesions [36]. By combining anatomical and functional information within a single imaging plane, integration of color, power, and spectral Doppler with the 3VT view improves diagnostic confidence and supports a more comprehensive assessment of fetal cardiovascular physiology [3,36,62].

### 7.2. Integration with 3D/4D Spatiotemporal Image Correlation (STIC)

Two-dimensional FE remains the foundation of prenatal cardiac assessment, whereas STIC provides complementary three- and four-dimensional (3D and 4D) visualization of cardiac anatomy and function [3,64]. By acquiring a volumetric dataset throughout the cardiac cycle, STIC enables offline analysis and multiplanar reconstruction of the fetal heart, facilitating detailed evaluation of complex cardiovascular structures [3,63,64,66,67]. When applied to the assessment of the outflow tracts and upper mediastinum, STIC may improve visualization of the spatial relationships among the great vessels and assist in characterizing complex conotruncal anomalies, including TGA [38,68]. Accordingly, 3D rendered images and multiplanar reconstructions enhance appreciation of vascular anatomy, vessel orientation, and arch morphology beyond what can be achieved with conventional 2D imaging alone [68,69]. STIC can also help visualize the connection between the *DA*, AoT, AoI, and DAo structures, which can sometimes be difficult to assess on a single 2D plane. In specialized centers, STIC enables remote expert review and tele-echocardiography, broadening access to advanced fetal cardiac evaluation [65]. Consequently, the integration of conventional 2D imaging and STIC provides complementary anatomical information that may improve the characterization of complex CHD and support prenatal counseling and perinatal planning [66,67,68,69].

### 7.3. Fetal Cardiac Function and Hemodynamic Implications Observed in the 3VT View

Contemporary FE extends beyond the detection of structural cardiac abnormalities and increasingly incorporates the assessment of fetal cardiovascular function and hemodynamics [3,20]. Within this context, the 3VT view represents a particularly informative imaging plane because it simultaneously depicts the PA, *DA*, AoT, SVC, and T, allowing evaluation of both anatomical relationships and flow dynamics within the upper mediastinum [8,21].

**Figure 6:**
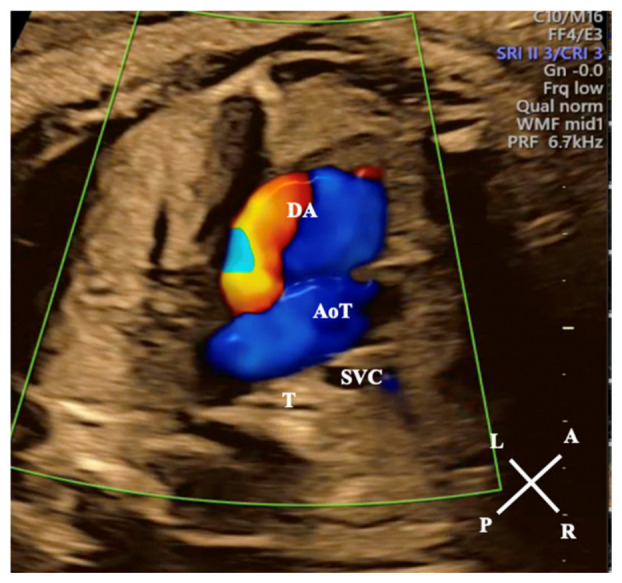
Echocardiographic transverse plane of the fetal thorax at the level of the classic 3VT view in a fetus with Pulmonary Atresia with intact interventricular septum. This view demonstrates, from left (L) to right (R), the ductal arch (*DA*) with reversed flow, the transverse aortic arch (AoT) with antegrade flow, and the superior vena cava (SVC).

Careful analysis of the 3VT view may reveal indirect signs of hemodynamic disturbance, including great-vessel disproportion, abnormal arch configuration, altered vessel alignment, and evidence of flow obstruction or abnormal shunting [1,3,14,42,44]. Integration of color and pulsed Doppler further refines this assessment by enabling characterization of flow direction, velocity, and turbulence within the great arteries and *DA* [36,70,71]. These findings may provide important insights into the physiological consequences of structural cardiac lesions and help identify fetuses at increased risk of ductal-dependent circulation or postnatal cardiovascular compromise [1,26,43] (Figure 6). Particular attention should be given to the relationship between the *DA*, AoT, and AoI, as this region represents a critical hemodynamic interface within the fetal circulation. Alterations in vessel caliber, flow distribution, or flow direction across this anatomical junction may reflect underlying abnormalities in arch development or cardiovascular adaptation and can provide valuable diagnostic information in selected CHD [49,70]. The integration of anatomical and functional information within a single transverse plane underscores the value of the 3VT view as a comprehensive tool for fetal cardiovascular assessment. Early recognition of hemodynamic abnormalities facilitates risk stratification, multidisciplinary counseling, delivery planning, and referral to centers with specialized neonatal cardiac expertise, thereby contributing to improved perinatal management and neonatal outcomes [1,20,69,71].

## 8. The 3VT View in Early Gestation

The 3VT view is well recognized as important during the mid-trimester scan, typically performed between 18 and 22 weeks of gestation [1,3]. However, growing evidence supports its use in the first trimester, when early identification of major CHD may facilitate timely referral and prenatal counseling [72,73].

### 8.1. Feasibility and Value of the 3VT View in the First Trimester

**Figure 7:**
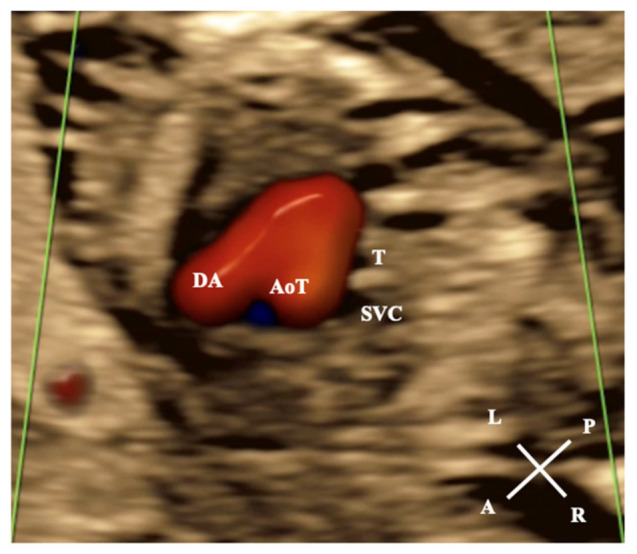
Echocardiographic transverse plane of the fetal thorax at the level of the classic 3VT view during a first-trimester scan with color Doppler interrogation. This view demonstrates, from left (L) to right (R), the ductal arch (*DA*) and the transverse aortic arch (AoT), both with antegrade flow, converging toward the descending aorta and forming the classic “V sign” to the left (L) of the trachea (T), followed by the superior vena cava (SVC).

The successful integration of any screening tool into routine clinical practice depends on its feasibility and reproducibility [1]. Early studies reported variable success rates in visualizing the great vessels during the first trimester, with image acquisition improving progressively as gestational age advanced [73]. However, advances in US technology, image resolution, and operator expertise have substantially improved the feasibility of early fetal cardiac assessment, including the systematic evaluation of the 3VT view [72,73]. Several studies have demonstrated that the 3VT plane can be consistently obtained during first-trimester examinations when performed by experienced operators. In a large prospective study of first-trimester fetal cardiac screening, Quarello et al. reported high feasibility rates for systematic cardiac assessment between 11 and 14 weeks’ gestation [72]. Similarly, Hutchinson et al. demonstrated that detailed visualization of fetal cardiac anatomy is achievable during the first trimester, particularly in specialized centers [73]. These findings support incorporating the 3VT view into early fetal cardiac assessment protocols [72,73]. Examinations are usually performed transabdominally using high-frequency probes, although a transvaginal approach may be useful when image quality is suboptimal [72,73]. The use of color Doppler is particularly valuable during early gestation because it facilitates the identification of the great vessels and improves visualization of flow patterns within the upper mediastinum [3,72,73]. A typical first-trimester 3VT view demonstrates the characteristic “V-sign,” formed by the convergence of the *DA* and AoT to the left of the trachea [8,14] (Figure 7). Despite the smaller fetal size and technical challenges inherent in early gestation, this anatomical configuration can usually be reliably identified, supporting the use of the 3VT view as an effective component of first-trimester fetal cardiac screening.

### 8.2. Role in Early Detection of Major Cardiac Anomalies and Chromosomal Risk Assessment

The principal value of the 3VT view during the first trimester lies in its ability to identify abnormalities of the great vessels and outflow tracts that may not be apparent on the 4CH view alone [8,14]. Several studies have demonstrated that incorporating the 3VT view into early fetal cardiac assessment improves the prenatal detection of major CHD and complements information obtained from conventional cardiac screening planes [21,72,74]. In a retrospective series of 1,596 pregnancies, the addition of the grayscale 3VT view increased the detection rate of CHD from 47.8% to 71.7%, with all major cardiac defects identified when the 4CH and 3VT views were evaluated together [21]. Similar results have been reported by other investigators, supporting the incorporation of the 3VT view into comprehensive first-trimester cardiac screening protocols [72]. Beyond overall detection rates, the 3VT view provides important diagnostic clues by assessing the number, size, alignment, and spatial relationships of the great vessels relative to the trachea [14]. One of the most relevant early findings is the single arterial vessel sign, characterized by the absence of the normal V-sign formed by the ductal and aortic arches [75]. Although initially associated with TGA, this sign has subsequently been reported in a broader spectrum of severe CHD, including hypoplastic left heart syndrome (HLHS), AoA obstruction, DORV, and CAT [74,75]. Quantitative assessment of vessel calibers may provide additional diagnostic information, as a disproportion between the PA and Ao can be associated with outflow-tract abnormalities, pulmonary stenosis, PAtr., TOF, and left-sided obstructive lesions [14]. Similarly, abnormalities of arch sidedness and vascular course can be identified early in gestation. A RAA, in which the arch courses to the right of the T, represents a readily recognizable finding within the 3VT plane and may indicate the presence of associated cardiovascular or extracardiac abnormalities [14,28]. Early assessment of the AoT and AoI may also provide valuable information regarding arch development, particularly in fetuses at risk of CoA or other left-sided obstructive lesions [76]. The 3VT view also contributes to prenatal risk assessment for genetic syndromes, particularly 22q11.2 deletion syndrome (22q11.2DS) [77,78]. Many of the cardiac defects most strongly associated with this microdeletion involve the great vessels and AoA, making the 3VT view especially useful for early recognition of affected fetuses. Findings such as RAA, IAA, and CAT should increase clinical suspicion and prompt consideration of genetic evaluation [77,78]. Integration of 3VT findings with other first-trimester markers, including increased nuchal translucency (NT), tricuspid regurgitation, and abnormal ductus venosus flow, may further refine prenatal risk stratification [67,76]. The incorporation of the 3VT view into first-trimester screening extends the benefits of transverse mediastinal assessment to an earlier stage of fetal development. Beyond improving the detection of major CHD, this plane provides important information regarding great-vessel anatomy, arch laterality, and early markers of genetic syndromes, supporting more comprehensive prenatal cardiovascular assessment and risk stratification [67,78,79,80,81].

## 9. The Concept of 3Y + T View

The 3VT view has become an integral component of contemporary fetal cardiac screening and is widely recognized as one of the most informative transverse planes for evaluating the upper mediastinum. Its standardized acquisition enables rapid assessment of the number, size, alignment, and spatial relationships of the great vessels and provides valuable diagnostic information regarding conotruncal defects, AoA abnormalities, and vascular rings. The hallmark of the conventional 3VT view is the characteristic “V-sign”, formed by the convergence of the *DA* and AoT to the left of the T. Although highly effective as a screening tool, the conventional interpretation of the 3VT view is primarily centered on recognition of this “V-sign”. Consequently, less emphasis is placed on the anatomical structures immediately distal to the point of convergence, particularly the AoI and the proximal DAo. This limitation may be relevant because the AoI occupies a unique position within the fetal circulation and plays a central role in several congenital cardiovascular abnormalities. The AoI represents the vascular segment located between the origin of the left subclavian artery and the insertion of the *DA*. From both anatomical and hemodynamic perspectives, it constitutes a watershed region where the outputs of the right and left ventricles interact before entering the DAo. As a result, alterations in ventricular loading conditions, arch development, or ductal flow frequently become evident at the isthmic level. Several clinically significant CHD directly involve this region, including CoA, aortic arch hypoplasia, IAA, HLHS, and other left-sided obstructive lesions. Consequently, careful evaluation of the AoI is fundamental for comprehensive assessment of fetal cardiovascular anatomy and function.

**Figure 8:**
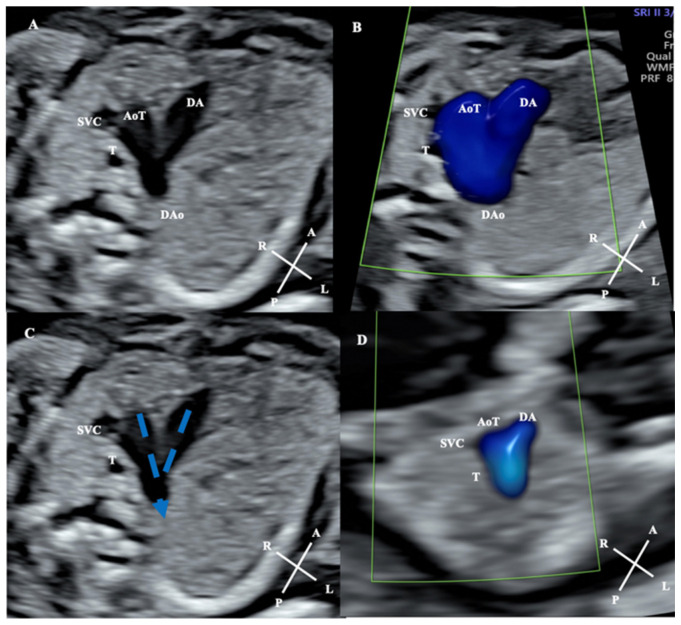
The proposed Three-Y and Trachea (3Y + T) view. The 3Y + T view is operationally defined as a transverse upper mediastinal plane derived from the conventional three-vessel and trachea (3VT) view in which the ductal arch (*DA*), transverse aortic arch (AoT), aortic isthmus (AoI), proximal descending aorta (DAo), and trachea (T) are simultaneously visualized within a single imaging plane. The view is obtained after identification of a standard 3VT plane, followed by a minimal cranial sweep and, when required, subtle transducer angulation or rotation to extend visualization beyond the conventional point of arch convergence. An adequate 3Y + T view requires simultaneous demonstration of the *DA* and AoT converging at the level of the AoI, continuity into the proximal DAo, and preservation of the T as a fixed anatomical landmark. In contrast to the conventional 3VT view, which primarily emphasizes the “V-sign”, the 3Y + T view focuses on the complete anatomical and Doppler-defined continuity between the *DA*, AoT, AoI, and DAo, creating a characteristic “Y-shaped” configuration. The DA and AoT form the two arms of the “Y”, whereas the AoI and proximal DAo constitute its stem. (**A**) Two-dimensional image demonstrating the 3Y + T plane. (**B**) Corresponding color Doppler image demonstrating uninterrupted antegrade flow from the *DA* and AoT toward the AoI and DAo. (**C**) Schematic representation of the 3Y + T configuration and its anatomical relationship to the T. (**D**) Example of the 3Y + T view obtained during a first-trimester examination.

The proposed Three-Y and Trachea (3Y + T) view is intended as a conceptual extension of the conventional 3VT view. Rather than focusing exclusively on the “V-sign”, the 3Y + T concept emphasizes the complete anatomical convergence of the *DA* and AoT into the AoI and proximal DAo. This creates a characteristic “Y-shaped” configuration in which the *DA* and AoT form the two arms of the “Y”, while the AoI and proximal DAo constitute its stem (Figure 8). Operationally, the 3Y + T view is defined as a transverse upper mediastinal plane derived from the conventional 3VT view in which the *DA*, AoT, AoI, and proximal DAo are simultaneously visualized in relation to the T. The view is obtained after identification of a standard 3VT plane, followed by a minimal cranial sweep and, when required, subtle transducer angulation or rotation to extend visualization beyond the conventional point of arch convergence. An adequate 3Y + T view requires simultaneous depiction of the *DA* and AoT converging at the level of the AoI, continuation into the proximal DAo, and preservation of the trachea within the same imaging plane. The trachea remains an essential anatomical landmark, preserving the orientation and laterality information that characterizes the standard 3VT view. From an anatomical perspective, the 3Y + T view provides a clearer demonstration of vascular continuity among the *DA*, AoT, AoI, and DAo. The *DA* is visualized as the continuation of the main PA, while the AoT courses alongside it before both vessels converge at the AoI and continue into the proximal DAo, thereby creating the characteristic “Y-shaped” configuration. The SVC remains positioned to the right of the arterial structures, and the trachea serves as a constant posterior landmark for evaluation of arch sidedness and mediastinal orientation. When color Doppler is applied, normal anatomy is characterized by an antegrade flow from both the *DA* and AoT toward the AoI and DAo, allowing simultaneous anatomical and functional assessment of arch convergence within a single imaging plane.

The proposed 3Y + T view should currently be regarded as a conceptual extension of the established 3VT examination rather than a validated diagnostic protocol. Its rationale is based on the hypothesis that a more cranial transverse plane may facilitate continuous visualization of the transverse aortic arch, aortic isthmus, ductal arch, and proximal descending aorta within a single imaging plane, potentially improving anatomical orientation and Doppler alignment. Although these theoretical advantages may enhance the assessment of left-sided obstructive lesions, particularly those involving the aortic arch and isthmus, the proposed concept is currently supported by anatomical rationale and repeated observational experience rather than prospective clinical evidence. Consequently, prospective studies are required to determine its reproducibility, feasibility, and incremental diagnostic value over the conventional 3VT view before routine clinical implementation. The potential value of the 3Y + T concept extends beyond anatomical visualization. By explicitly incorporating the AoI into the assessment, this approach may facilitate the evaluation of arch continuity, isthmic caliber, and the spatial relationship between the systemic and ductal circulations. Such information may be particularly relevant in conditions characterized by abnormal development of the AoA, including CoA, IAA, and other obstructive left-sided lesions. Color Doppler interrogation plays a central role in the proposed 3Y + T view. In addition to confirming normal vascular continuity, Doppler imaging may facilitate assessment of flow direction and relative flow distribution through the *DA*, AoA, and AoI. Simultaneous visualization of these structures may allow more targeted Doppler sampling and potentially improve characterization of hemodynamic abnormalities in selected CHD. Furthermore, preserving the trachea within the imaging plane maintains the diagnostic advantages of the conventional 3VT view in evaluating vascular laterality and abnormal arch configurations. As a conceptual extension of the established 3VT view, the proposed 3Y + T approach seeks to shift attention from simple recognition of the “V-sign” toward a more integrated evaluation of arch convergence, vascular continuity, and isthmic anatomy (Table 1). Rather than replacing the conventional 3VT view, the 3Y + T concept is intended to complement existing fetal cardiac screening protocols by highlighting anatomical and hemodynamic information that may otherwise receive less emphasis during routine examinations.

**Figure 9:**
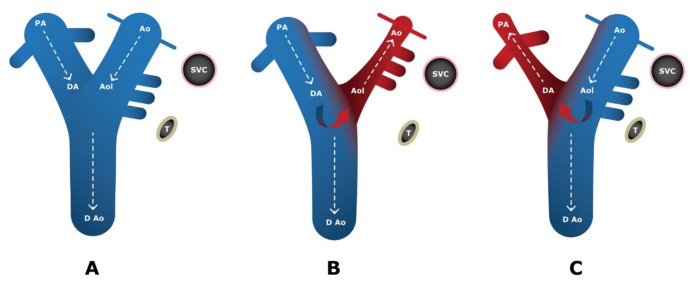
Conceptual comparison between the conventional three-vessel and trachea (3VT) view and the proposed Three-Y and Trachea (3Y + T) view. The conventional 3VT plane emphasizes the transverse visualization of the great vessels and the characteristic “V-sign” formed by the convergence of the ductal arch (*DA*) and transverse aortic arch (AoT). The proposed 3Y + T view is derived from the conventional 3VT plane through a minimal cranial sweep and, when required, subtle transducer angulation or rotation, extending visualization beyond the conventional point of arch convergence to incorporate the aortic isthmus (AoI) and proximal descending aorta (DAo) within the same imaging plane. This conceptual extension emphasizes arch continuity, isthmic morphology, and the anatomical and hemodynamic relationship between the systemic and ductal circulations. The resulting “Y-shaped” configuration highlights the central role of the AoI as the junction between the *DA*, AoT, and DAo, providing a framework for integrated anatomical and Doppler assessment of the fetal upper mediastinum. In normal anatomy, the *DA* and AoT demonstrate concordant antegrade flow and converge into the DAo to the left of the trachea (T), forming the characteristic “Y” configuration (**A**). In a fetus with severe left-sided obstructive disease (**B**), the 3Y + T view facilitates visualization of a hypoplastic AoT and AoI and may demonstrate retrograde flow from the *DA* toward the AoT. In a fetus with severe right-sided obstructive disease (**C**), the 3Y + T view may demonstrate retrograde flow from the AoT toward the *DA* and pulmonary artery (PA), which is frequently smaller than the AoT. SVC = superior vena cava.

The 3Y + T view is presented here as a conceptual framework for fetal cardiac assessment. Its feasibility, reproducibility, and potential diagnostic contribution beyond the conventional 3VT view require prospective validation. Future studies should establish standardized acquisition criteria, define normative measurements for the AoI and related vascular structures throughout gestation, evaluate interobserver reproducibility, and determine whether incorporation of the 3Y + T concept improves detection of specific congenital cardiovascular abnormalities (Figure 8). If validated, this approach may represent a useful addition to contemporary fetal echocardiographic assessment of the upper mediastinum (Figure 9).

This table compares the main anatomical orientation, imaging focus, distal arch visualization, and Doppler alignment features of the conventional 3VT view with the proposed 3Y + T view. The 3Y + T approach expands on the traditional transverse 3VT plane by adding longitudinal visualization of the aortic isthmus and proximal descending aorta, potentially improving anatomical and hemodynamic evaluation.

## 10. Limitations and Challenges

### 10.1. Factors Affecting Visualization

Since the 3Y + T concept represents a newly proposed extension of upper mediastinal fetal cardiac imaging, no published studies have specifically evaluated its acquisition, reproducibility, or clinical applicability. Consequently, considerations regarding its technical limitations, operator dependence, and standardization must currently be extrapolated from the extensive literature on the 3VT view and outflow-tract imaging, which share similar anatomical and methodological foundations. Visualization of the upper mediastinal great vessels relies on the acquisition of standardized transverse planes, particularly the 3VT view, which is incorporated into contemporary international screening guidelines [1,3]. Because the proposed 3Y + T concept is derived from the same upper mediastinal imaging approach as the conventional 3VT view, many of the technical challenges associated with 3VT acquisition are likely to apply to its implementation. However, consistent visualization of this anatomical region is influenced by several technical and biological factors. Fetal position and movement may hinder stable acquisition of the optimal transverse plane, affecting assessment of the great-vessel relationships. Maternal habitus may further reduce image quality by increasing ultrasound attenuation and imaging depth, thereby limiting visualization of mediastinal vascular structures. Gestational age also influences image quality, as early examinations are constrained by the small caliber of the great vessels and reduced spatial resolution of the outflow tracts [72,73,82]. In addition, minor probe angulation errors may result in incomplete visualization of the vascular structures required for detailed assessment of the upper mediastinum [14,83]. Color Doppler is often necessary to confirm vessel identity and flow direction, particularly when 2D imaging is suboptimal [1,3]. Even among experienced operators, incomplete acquisition of the 3VT view has been reported, emphasizing the importance of adequate training and technical expertise [14,72,83]. These limitations are inherent to upper mediastinal imaging and are therefore likely to affect detailed assessment of the anatomical relationships emphasized by the proposed 3Y + T concept. Practical measures such as transducer repositioning, maternal repositioning, and repeat examination may help overcome technical difficulties and improve image acquisition [1].

### 10.2. Interobserver Variability and Training Needs

FE remains highly operator-dependent, and interobserver variability persists despite the widespread adoption of standardized cardiac screening protocols [83]. Studies evaluating the reproducibility of fetal cardiac examinations have demonstrated that the acquisition and interpretation of outflow-tract and great-vessel views are influenced by operator experience, technical expertise, and adherence to standardized imaging methodologies [1,83]. Although the 3VT view is now an established part of routine fetal cardiac screening, accurate acquisition and interpretation require precise probe manipulation and a thorough understanding of upper mediastinal anatomy [1,14]. Assessment of great vessel relationships, arch sidedness, and vascular configuration may therefore vary depending on the examiner’s level of experience, especially in challenging clinical situations [17,83]. Structured training programs focused on outflow-tract imaging, great-vessel anatomy, and transverse mediastinal assessment have been shown to improve diagnostic performance and increase the reproducibility of fetal cardiac examinations [17,83,84]. Simulation-based education, standardized acquisition protocols, and competency-based assessment may further enhance operator confidence and consistency in clinical practice [84]. Because the proposed 3Y + T concept emphasizes detailed evaluation of the spatial relationship among the ductal arch, AoT, AoI, and DAo, dedicated training and standardized acquisition criteria are likely to be important for its reliable implementation [1]. Future studies evaluating interobserver agreement and reproducibility will be essential to determine the feasibility and clinical applicability of this proposed imaging approach [1,16].

### 10.3. Need for Standardized Reporting Criteria

International guidelines emphasize the importance of standardized imaging protocols and structured documentation for fetal cardiac screening [1,3]. The incorporation of the 3VT view into routine clinical practice has significantly improved prenatal detection of outflow-tract and aortic arch abnormalities, demonstrating the value of consistent image acquisition and reporting standards [14,21]. Despite these advances, variability in acquisition techniques, image quality, and reporting terminology continues to affect reproducibility across institutions and studies [1,3]. Standardized documentation of imaging planes, Doppler findings, and anatomical relationships is therefore essential for quality assurance, interobserver consistency, and effective multidisciplinary communication [3]. Uniform reporting also facilitates comparison of results across centers and supports the development of evidence-based screening protocols. By extension, the absence of consensus criteria for the acquisition and interpretation of novel or extended upper mediastinal imaging approaches may limit both clinical implementation and research validation. For the proposed 3Y + T concept, standardization would ideally include consistent definitions of vessel alignment, arch configuration, aortic isthmus morphology, patterns of ductal–aortic convergence, and associated flow characteristics. Establishing minimum imaging requirements and uniform terminology would be expected to improve reproducibility and facilitate multicenter evaluation. Ultimately, standardization is essential not only for methodological consistency but also for effective clinical implementation, as uniform reporting influences screening performance, referral pathways, and diagnostic accuracy [1,3]. In this context, the proposed 3Y + T concept may represent a logical extension of conventional 3VT imaging, although dedicated validation and reproducibility studies will be required before its potential clinical role can be fully established. Importantly, no prospective studies have yet demonstrated that the 3Y + T concept improves diagnostic performance or provides incremental clinical value beyond the conventional 3VT view.

## 11. Future Perspectives

### 11.1. AI-Assisted Acquisition and Interpretation

Artificial intelligence (AI) is increasingly being integrated into fetal cardiac imaging workflows, with applications ranging from image acquisition and quality assessment to automated view recognition and diagnostic support [85,86,87]. This evolution aligns with international screening recommendations that emphasize the importance of consistently obtaining standardized cardiac views, including the 3VT view, for effective prenatal detection of CHD [1]. Recent deep-learning (DL) systems have demonstrated the ability to automatically identify key fetal cardiac screening views, including the 3VT plane, before diagnostic interpretation [85]. This capability is clinically relevant because incomplete acquisition of essential cardiac views and inconsistent adherence to standardized protocols remain major limitations of fetal cardiac screening outside specialized centers [1,85]. Automated view recognition and image-quality assessment may therefore help reduce operator-dependent variability and improve the consistency of screening examinations [85,86]. Prospective validation studies have further shown that AI algorithms can distinguish diagnostic from nondiagnostic fetal echocardiographic images and provide real-time quality feedback, potentially prompting image reacquisition when required [86]. Such feedback mechanisms may support a more standardized and iterative acquisition process, reducing operator dependence and improving examination reliability in routine clinical practice [86]. Complementary approaches such as Fetal Intelligent Navigation Echocardiography (FINE) extend this concept by applying intelligent navigation to STIC datasets, automatically generating and displaying standardized diagnostic cardiac planes [87]. By reducing reliance on manual plane selection and facilitating visualization of complex anatomical relationships, these technologies may improve reproducibility and workflow efficiency [87]. Collectively, these developments suggest that AI may increasingly contribute not only to diagnostic interpretation but also to acquisition guidance, quality assurance, and workflow standardization, thereby supporting broader implementation of high-quality prenatal cardiac screening [85,86,87].

### 11.2. Automated Detection of Abnormal 3VT Anatomy

The 3VT view provides a standardized representation of the great vessels, T, and upper mediastinal anatomy, making it a particularly attractive target for automated image analysis and machine-learning–based pattern recognition [1,85]. Because this view simultaneously depicts vessel number, size, alignment, arch sidedness, and their relationship to the T, it contains many of the anatomical features used in the prenatal detection of conotruncal and AoA abnormalities [1,14]. Recent AI-based approaches have demonstrated high diagnostic performance in identifying complex CHD from standardized fetal cardiac imaging datasets that include the 3VT plane [85]. These developments suggest that future automated screening systems may be capable of recognizing abnormal anatomical patterns within selected cardiac views, potentially allowing rapid triage of examinations that require expert review. Such systems could facilitate earlier referral of high-risk cases while reducing the burden of manual image interpretation in large-scale screening programs [85,86]. Reliable automation, however, depends not only on pattern recognition but also on image quality. Recent studies have shown that segmentation and classification performance declines when imaging planes are incomplete or poorly acquired, highlighting the importance of integrating quality-control algorithms into automated workflows [86]. Consequently, future AI systems are likely to combine automated view recognition, image-quality assessment, and diagnostic classification within a unified screening framework. Advances in intelligent navigation and volumetric imaging further support this direction by enabling standardized reconstruction of cardiac planes and more systematic evaluation of fetal cardiovascular anatomy [87]. Together, these developments suggest that the 3VT view may become an important interface between conventional fetal cardiac screening and future AI-assisted diagnostic pathways.

### 11.3. Role of the 3VT View in Fetal Cardiac Telemedicine and Screening Program

Tele-echocardiography has become an increasingly important component of fetal cardiac assessment, enabling remote image review, specialist consultation, and expert interpretation in regions with limited access to fetal cardiology services. Advances in digital infrastructure, image transmission, and workflow integration have further expanded its clinical applicability, demonstrating feasibility, diagnostic utility, and patient acceptance across diverse healthcare settings [88,89]. The 3VT view is particularly well suited for tele-screening because of its standardized acquisition, reproducibility, and diagnostic value in the assessment of conotruncal and aortic arch abnormalities [1,3]. As one of the key planes recommended by international screening guidelines, it can be acquired by trained sonographers and subsequently reviewed remotely by fetal cardiology specialists, thereby facilitating access to expert evaluation while maintaining standardized screening pathways [1,3]. Future screening programs may incorporate hybrid models in which local operators acquire protocol-driven fetal cardiac datasets, including the 3VT view, while AI systems provide real-time image quality assessment and preliminary triage [85,86]. Examinations identified as abnormal, incomplete, or diagnostically uncertain could then be referred for remote expert review. Such an approach has the potential to improve resource allocation, reduce operator-dependent variability, and expand access to specialized fetal cardiac assessment [85,86,88]. The integration of telemedicine, AI-assisted imaging, and standardized screening protocols may therefore represent a scalable strategy for improving prenatal detection of CHD and strengthening referral networks between primary screening centers and specialized fetal cardiology units [1,3,86,88]. At present, however, no validated artificial intelligence systems have been specifically developed for the proposed 3Y + T concept. Although AI-assisted acquisition and interpretation tools have been successfully applied to the 3V and 3VT views because of their standardized acquisition and clearly defined anatomical landmarks [85,86,87], the proposed 3Y + T concept represents a more complex anatomical framework. Rather than a single fixed imaging plane, it emphasizes the spatial relationships among the ductal arch, AoT, AoI, and DAo, requiring integrated anatomical and hemodynamic interpretation. Consequently, dedicated datasets, standardized acquisition criteria, and prospective validation studies will be necessary before AI-assisted implementation of the 3Y + T concept can be realistically considered. Beyond structural assessment, fetal cardiovascular imaging is evolving toward comprehensive cardiovascular phenotyping by integrating quantitative functional imaging. Two-dimensional speckle-tracking echocardiography has emerged as a promising technique for assessing fetal myocardial deformation, providing complementary information on myocardial function in fetuses with CHD and other high-risk pregnancies [90,91]. Future integration of the proposed 3Y + T concept into multimodal fetal cardiovascular imaging frameworks, incorporating myocardial deformation imaging, STIC, 3D and 4D imaging, AI-assisted image analysis, and quantitative flow assessment, may further enhance comprehensive fetal cardiovascular assessment and represent an important direction for future research.

## 12. The Aortic Isthmus as a Central Anatomical and Hemodynamic Landmark

The AoI holds a distinctive position in fetal circulation, marking the anatomical junction between the systemic and ductal circulations [90]. Positioned between the origin of the left subclavian artery and the insertion of the DA, it functions as a hemodynamic watershed where outputs from the left and right ventricles converge before entering the DAo. Consequently, changes in ventricular loading conditions, vascular resistance, arch development, and ductal flow patterns are reflected at the AoI level [92,93]. The clinical significance of this vascular segment goes beyond its anatomical position. Several congenital heart abnormalities directly involve the AoI or its neighboring structures, including CoA, AoA hypoplasia, IAA, HLHS, and other types of left-sided obstructive disease [14,26]. In addition, Doppler interrogation of the AoI has been used to investigate fetal cardiovascular adaptation in conditions such as fetal growth restriction, placental insufficiency, and altered fetal hemodynamics [93,94,95]. Despite its recognized importance, the AoI is not usually the primary focus of conventional interpretations of the 3VT view, which remain largely centered on identifying the characteristic V-sign, vessel arrangement, and AoA laterality [1,8].

The proposed 3Y + T concept shifts attention to the region where the *DA*, AoT, AoI, and DAo converge, emphasizing a vascular segment that may provide additional insights into vascular anatomy and flow relationships. Whether systematic visualization of this region ultimately improves diagnostic performance remains unknown and requires prospective investigation. Nevertheless, the established physiological significance of the AoI provides a strong scientific rationale for exploring imaging frameworks that facilitate its assessment within routine FE [92,93]. By explicitly incorporating the AoI and proximal DAo into a standardized upper mediastinal imaging plane, the 3Y + T concept can improve the overall evaluation of arch continuity, vascular convergence, and flow dynamics in fetal circulation.

## 13. Conclusions

The 3VT view has become a cornerstone of contemporary FE, providing a rapid, reproducible, and standardized assessment of the great vessels and their relationship to the trachea within a single transverse mediastinal plane. When integrated with the 4CH and outflow-tract views, it substantially improves prenatal detection of major CHD, particularly conotruncal anomalies, AoA abnormalities, and vascular rings that may be overlooked with conventional cardiac screening planes alone. Beyond its established role as a screening tool, the 3VT view has evolved into a comprehensive imaging approach that integrates anatomical and functional assessment using color Doppler, spectral Doppler, volumetric imaging, and STIC technologies. These developments have further strengthened its value in contemporary fetal cardiac evaluation.

The proposed Three-Y and Trachea (3Y + T) concept is an extension of the conventional 3VT view. By emphasizing the anatomical convergence of the *DA* and AoT at the AoI and their continuation into the DAo, the 3Y + T approach promotes a more integrated assessment of arch continuity, isthmic morphology, and upper mediastinal vascular relationships. Given the central anatomical and hemodynamic role of the AoI in the fetal circulation, this perspective may offer additional anatomical insights into conditions affecting the AoA and ductal circulation, including CoA, AoA hypoplasia, IAA, and other severe right- or left-sided obstructive lesions. The 3Y + T view should currently be regarded as a conceptual framework intended to complement, rather than replace, the conventional 3VT examination. Its feasibility, reproducibility, and potential diagnostic contribution remain to be established through prospective validation studies. Future research should define standardized acquisition criteria, establish normative reference data, evaluate interobserver reproducibility, and determine whether incorporating the 3Y + T concept yields measurable benefits in fetal cardiac assessment. As FE continues to evolve through advances in AI-assisted imaging, automated view recognition, advanced Doppler technologies, and tele-echocardiography, the 3Y + T concept may provide an additional framework for exploring the anatomical and functional relationships of the fetal upper mediastinum and AoA.

## Figures and Tables

**Table 1:** Structural and functional framework distinguishing the 3VT and 3Y + T echocardiographic views.

Feature	3VT	3Y + T
**Primary landmark**	Trachea	Trachea
**Main anatomical focus**	Vessel arrangement	**Vessel convergence**
**Aortic isthmus visualization**	Limited	**Enhanced**
**Descending aorta visualization**	Partial	**Explicit**
**Assessment of arch continuity**	Limited	**Enhanced**
**Functional Doppler interrogation**	General	**More targeted**
**Conceptual emphasis**	V-sign	**Y-sign**

## Data Availability

No new data were created or analyzed in this study. Data sharing is not applicable to this article.

## Abbreviations

The following abbreviations are used in this manuscript:

22q11.2DS	22q11.2 deletion syndrome
2D	Two-dimensional
3D	Three-dimensional
3V view	Three-vessel view
3VT view	Three-vessel and trachea view
3Y + T	Three-Y and Trachea view
4D	Four-dimensional
4CH view	Four-chamber view
AAo	Ascending aorta
AI	Artificial intelligence
Ao	Aorta
AoA	Aortic arch
AoI	Aortic isthmus
AoT	Transverse aortic arch
ARSA	Aberrant right subclavian artery
CAT	Common arterial trunk
CHD	Congenital heart disease
CoA	Coarctation of the aorta
DA	Ductus arteriosus
DAA	Double aortic arch
DAo	Descending aorta
DL	Deep learning
DORV	Double-outlet right ventricle
FE	Fetal echocardiography
FINE	Fetal Intelligent Navigation Echocardiography
HLHS	Hypoplastic left heart syndrome
IAA	Interrupted aortic arch
ISUOG	International Society of Ultrasound in Obstetrics and Gynecology
LV	Left ventricle
LVOT	Left ventricular outflow tract
MeSH	Medical Subject Headings
NT	Nuchal translucency
PA	Pulmonary artery
PAtr.	Pulmonary atresia
PA-VSD	Pulmonary atresia with ventricular septal defect
PLSVC	Persistent left superior vena cava
PRF	Pulse repetition frequency
PRISMA	Preferred Reporting Items for Systematic Reviews and Meta-Analyses
RAA	Right aortic arch
RVOT	Right ventricular outflow tract
STIC	Spatiotemporal image correlation
SVC	Superior vena cava
T	Trachea
TGA	Transposition of the great arteries
TOF	Tetralogy of Fallot
US	Ultrasound
VSD	Ventricular septal defect
